# Diagnostic and Prognostic Evaluation of Disseminated Intravascular Coagulation Using the Disseminated Intravascular Coagulation Index

**DOI:** 10.3390/jcm14217478

**Published:** 2025-10-22

**Authors:** Eri Nakano, Hideo Wada, Akitaka Yamamoto, Masaki Tomida, Yuhuko Ichikawa, Katsuya Shiraki, Motomu Shimaoka, Hideto Shimpo, Isao Tawara

**Affiliations:** 1 Department of Hematology and Oncology, Mie University Graduate School of Medicine, Tsu 514-8507, Japan; e.nakano12@gmail.com (E.N.); itawara@clin.medic.mie-u.ac.jp (I.T.); 2 Department of Hematology, Suzuka Kaisei Hospital, Suzuka 513-0836, Japan; 3 Department of General Medicine, Mie Prefectural General Medical Center, Yokkaichi 510-8561, Japan; katsuya-shiraki@mie-gmc.jp; 4 Department of Emergency and Critical Care Center, Mie Prefectural General Medical Center, Yokkaichi 510-8561, Japan; akitaka-yamamoto@mie-gmc.jp (A.Y.); masaki-tomida@mie-gmc.jp (M.T.); 5 Department of Central Laboratory, Mie Prefectural General Medical Center, Yokkaichi 510-8561, Japan; ichi911239@yahoo.co.jp; 6 Department of Molecular Pathobiology and Cell Adhesion Biology, Mie University Graduate School of Medicine, Tsu 514-8507, Japan; motomushimaoka@gmail.com; 7 Mie Prefectural General Medical Center, Yokkaichi 510-8561, Japan; hideto-shimpo@mie-gmc.jp

**Keywords:** DIC, DIC index, PT-INR, platelet count, D-dimer

## Abstract

**Background**: Diagnostic criteria for disseminated intravascular coagulation (DIC) have been established by the Japanese Ministry of Health, Labor, and Welfare (JMHLW), the International Society of Thrombosis Hemostasis (ISTH), and the Japanese Association for Acute Medicine (JAAM). These criteria vary and are complicated, and the cutoff values differ, so a simple and rapid diagnostic approach for DIC is needed. **Materials and Methods**: The usefulness of the DIC index (prothrombin time-international normalized ratio [PT-INR] x D-dimer/platelet count) for diagnosing DIC and predicting outcomes in 1500 critically ill patients was assessed. **Results**: The PT-INR, D-dimer level, and DIC index were significantly higher in patients with DIC than in those without DIC, and their platelet count was significantly lower. Receiver operating characteristic (ROC) analyses showed that the diagnostic agreement was the highest for the JMHLW score among the three diagnostic criteria. The PT-INR, D-dimer level, DIC index, and JMHLW, ISTH overt-DIC, and modified JAAM DIC scores were significantly higher in non-survivors than in survivors, and their platelet counts were significantly lower. Although ROC analyses showed that the PT-INR, D-dimer level, platelet count, DIC index, JMHLW, ISTH overt-DIC, and modified JAAM DIC scores were related to the outcome, the cutoff values of the DIC index, and JMHLW, ISTH overt-DIC and modified JAAM DIC scores were low. **Conclusions**: The DIC index was highly consistent with the three diagnostic criteria for DIC and related outcomes.

## 1. Introduction

Disseminated intravascular coagulation (DIC) is often associated with organ failure due to thrombosis and bleeding, resulting in high mortality rates [1,2,3]. The underlying causes of DIC include infectious diseases [4,5], hematological malignancy [6], solid cancer [7], obstetrics [8], trauma [9], and aneurysms [10]. Although physiological protease inhibitors, including antithrombin and recombinant thrombomodulin, and the treatment of underlying diseases have been recommended for the treatment of DIC patients in Japan [11,12], the treatment of underlying diseases and the administration of supplementary therapy from blood products for major bleeding in DIC patients are mainly recommended in Europe and North America [13,14,15].

Differentiating DIC from thrombotic microangiopathy (TMA) is difficult [16,17]. Furthermore, there is no specific biomarker for diagnosing DIC; for example, platelet counts, prothrombin time (PT), the PT-international normalized ratio (INR), fibrin-related markers including the D-dimer level, soluble fibrin, fibrinogen, and fibrin degradation products (FDP), and antithrombin are not specific biomarkers of DIC. Therefore, DIC diagnostic criteria that use a scoring system based on the above biomarkers or clinical symptoms have been established by the Japanese Ministry of Health, Labor, and Welfare (JMHLW) [18], the International Society of Thrombosis Hemostasis (ISTH) [19], the Japanese Association for Acute Medicine (JAAM) [20], and the Japanese Society of Thrombosis Hemostasis [21]. In addition, sepsis-induced coagulopathy (SIC) [22,23,24] has been subsequently observed. However, the mortality associated with this pathology is different among DIC patients when various diagnostic criteria are used [25]; this has resulted in several of the DIC guidelines used to evaluate the efficacy of treatments indicating a different outcome in DIC patients. Determining the difference in mortality among several DIC groups using different diagnostic criteria may be complicated for physicians. The standardization of DIC diagnostic criteria or a uniform index may therefore be required in order to evaluate the severity of DIC.

A scoring system using several biomarkers for diagnosing DIC [18,19,20,21] may be complicated in practice and fibrin-related markers require standardization [26,27]. Simple diagnostic criteria are required to facilitate the early and prompt treatment of DIC in the emergency room or intensive care unit. The SIC score [22,23,24] includes the PT, platelet count, and the sequential organ failure assessment score. We have also proposed a rapid scoring [28] system for the diagnosis of DIC that uses adequate cutoff values for PT, D-dimer level, and platelet count. However, the cutoff values of the parameters varied among the four diagnostic criteria for DIC [18,19,20,21], suggesting that the cutoff value and score for each parameter may not be clear to physicians. Therefore, we created the following super formula for diagnosing DIC: soluble C-type lectin-like receptor 2 (sCLEC-2) level × D-dimer level/platelet count [29]. Although sCLEC-2 is a useful biomarker for platelet activation [30,31], it is not a common biomarker in general hospitals.

In the present study, we examined the utility of a DIC index formula that substituted the PT-INR (international normalized ratio) for the sCLEC-2 level in the super formula for diagnosing DIC and compared it with the JMHLW diagnostic criteria for DIC. The mortality was lowest in those using JAAM diagnostic criteria for DIC and highest in those using ISTH diagnostic criteria for DIC [25], suggesting that using the JMHLW diagnostic criteria for DIC was more appropriate for this study.

## 2. Materials and Methods

The study population included 1500 consecutive samples in which the PT-INR, platelet count, and D-dimer values were measured; these samples were obtained from patients with the following conditions who were managed at Mie Prefectural General Medical Center from 1 November 2019 to 28 December 2024: infectious disease (*n* = 289), digestive system disease (*n* = 88), chest or abdominal aneurysm (*n* = 86), hematological malignancy (*n* = 67), trauma (*n* = 67), obstetrics disease (*n* = 51), respiratory disease (*n* = 38), arrhythmia (*n* = 21), thrombosis of the peripheral artery (*n* = 16), heat illness (*n* = 35), cardiac pulmonary arrest (CPA, *n* = 67), solid cancer (*n* = 36), acute coronary syndrome (ACS, *n* = 72), heart failure (*n* = 86), convulsive disorder (*n* = 26), metabolic and endocrine diseases (*n* = 20), venous thromboembolism (*n* = 43), cerebral bleeding (n = 58), non-malignant hematological disease (*n* = 38), cerebral thrombosis (*n* = 145), indefinite compliant syndrome (*n* = 88), and others (*n* = 61). DIC was diagnosed using the JMHLW criteria for DIC [18]; patients with DIC scores of ≥7 points, 5–6 points, and ≤4 points were diagnosed with JMHLW-DIC, JMHLW-Pre-DIC, and JMHLW-Non-DIC, respectively (Table 1 and Table 2). Patients who did not agree to participate in the study, or did not have the above measurements or sufficient clinical records, were excluded. DIC in these patients was also evaluated using the ISTH overt DIC criteria [19] or the modified JAAM (mJAAM) criteria for DIC [20] without point of systemic inflammatory response syndrome (SIRS) [32]; patients with ISTH overt DIC scores of ≥5 points and ≤4 points were diagnosed with ISTH-DIC and ISTH-Non-DIC, respectively, and patients with mJAAM DIC scores of ≥4 points, ≥3 points, and ≤2 points were diagnosed with mJAAM-DIC (≥4), mJAAM-DIC (≥3), and mJAAM-Non-DIC, respectively. The DIC index was calculated using the following formula: DIC index = PT-INRxD-dimer/platelet count (Table 3). The study protocol (2019-K9) was approved by the Human Ethics Review Committee of the Mie Prefectural General Medical Center, and informed consent was obtained from each participant. This study was conducted in accordance with the principles of the Declaration of Helsinki.

D-dimer and FDP levels were measured using the LPIA-Genesis and LPIA-FDP-P, respectively (PHC Co, Tokyo, Japan), using a STACIA system (PHC Co). The PT-INR value and fibrinogen levels were measured using Thromborel S and Thrombin reagent LQ, respectively (Sysmex Co., Kobe, Japan), with an automatic coagulation analyzer CS-5100 (Sysmex Co). Platelet counts were measured using a fully automatic blood cell counter—XN-3000 (Sysmex Co.).

### Statistical Analyses

Data are expressed as the median (25th–75th percentile). The significance of the differences between groups was examined using the Mann–Whitney *U*-test. The cut-off values were examined using a receiver operating characteristic (ROC) analysis [33].

Statistically, significance was set at *p* < 0.05. All statistical analyses were performed using the Stat-Flex software program (version 6; Artec Co., Ltd., Osaka, Japan).

## 3. Results

In this study, the underlying diseases varied, and the median age was high among the DIC, pre-DIC, and non-DIC groups (Table 2). The percentage of patients with JMHLW-DIC and JMHLW-Pre-DIC were 4.8% and 12.3%, respectively. The percentage of those with ISTH-DIC, mJAAM (≥4), and those with mJAAM (≥3) was 4.7%, 8.5%, and 15.6%, respectively.

**Figure 1:**
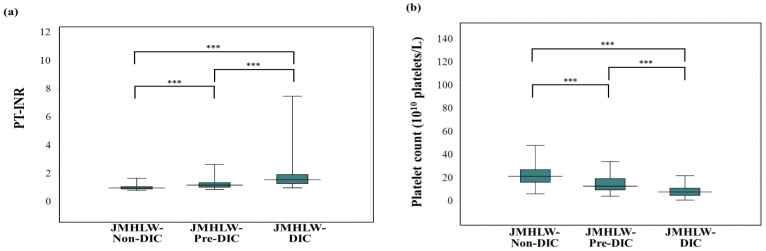

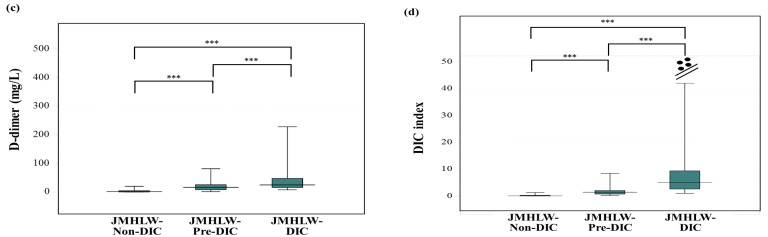
Box-and-whisker plot of the PT-INR: (**a**) platelet count, (**b**) D-dimer levels, and (**c**) DIC index (**d**) among JMHLW-Non-DIC, JMHLW-Pre-DIC, and JMHLW-DIC. PT, prothrombin time; INR, international normalized ratio; DIC, disseminated intravascular coagulation; JMHLW, Japanese Ministry of Health, Labor, and Welfare; patients with DIC scores of ≥7 points, 5–6 points, and ≤4 points were diagnosed with JMHLW-DIC, JMHLW-Pre-DIC, and JMHLW-Non-DIC, respectively. *** *p* < 0.001. ●●●●// > 5.0.

Regarding the relationship between biomarkers and the JMHLW DIC score, PT-INR (median; 25th–75th percentile) was significantly higher in those with JMHLW-DIC (1.59; 1.33–1.96) than in those with JMHLW-Pre-DIC (1.21; 1.06–1.38) or JMHLW-Non-DIC (1.03; 0.95–1.15), and higher in those with JMHLW-Pre-DIC than in those with JMHLW-Non-DIC a ≤4 (Figure 1a). The platelet count was significantly lower in those with JMHLW-DIC (8.1 × 10^10^ platelets/L; 5.2–11.4 × 10^10^ platelets/L) than in those with JMHLW-Pre-DIC (11.0 × 10^10^ platelets/L; 7.7–15.7 × 10^10^ platelets/L) or JMHLW-Non-DIC (21.6 × 10^10^ platelets/L; 16.4–27.5 × 10^10^ platelets/L), and lower in those with JMHLW-Pre-DIC than in those with JMHLW-Non-DIC (Figure 1b). The D-dimer levels were significantly higher in those with JMHLW-DIC (25.0 mg/L; 16.1–47.8 mg/L) than in those with JMHLW-Pre-DIC (17.0 mg/L; 8.2–25.7 mg/L) or JMHLW-Non-DIC (1.7 mg/L; 0.7–4.8 mg/L) and higher in those with JMHLW-Pre-DIC than in those with JMHLW-Non-DIC (Figure 1c). Regarding the relationship between the DIC index and JMHLW DIC score, the DIC index was significantly higher in those with JMHLW-DIC (6.10; 2.73–13.10) than in those with JMHLW-Pre-DIC (1.44; 0.74–2.10) or JMHLW-Non-DIC (0.09; 0.03–0.27) and higher in those with JMHLW-Pre-DIC than in those with JMHLW-Non-DIC (Figure 1d). The correlation coefficients of the PT-INR, 1/platelet count, and D-dimer level with the JMHLW DIC score were 0.449 (*p* < 0.001), 0.159 (*p* < 0.001), and 0.542 (*p* < 0.001), respectively. The correlation coefficient of the DIC index with the JMHLW DIC score was 0.192 (Y = −7.084 + 6.021X, *p* < 0.001) (Table 4).

ROC analyses (JMHLW-DIC vs. JMHLW-Non-DIC) showed that the DIC index was markedly more useful for diagnosing DIC than the PT-INR, platelet count, and D-dimer level, and that the DIC index was more useful for diagnosing DIC than for the PT-INR/Platelet count, PT-INRxD-dimer, and D-dimer/Platelet count level (Table 5). ROC analyses (JMHLW-DIC or JMHLW-Pe-DIC vs. JMHLW-Non-DIC) showed that the DIC index was markedly more useful for diagnosing DIC than the PT-INR, platelet count, D-dimer level, PT-INR/Platelet count, PT-INRxD-dimer, and D-dimer/Platelet count level.

**Figure 2:**
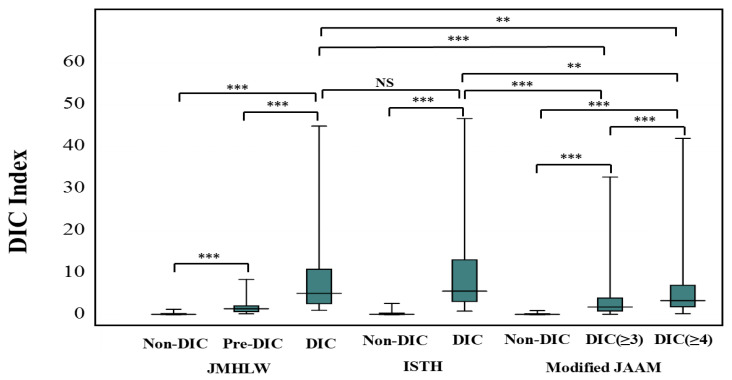
DIC index in the patients with JMHLW-Non-DIC, JMHLW-Pre-DIC, ISTH Non-DIC, ISTH-DIC, mJAAM-Non-DIC, mJAAM-DIC (≥3), and mJAAM-DIC (≥4). DIC, disseminated intravascular coagulation; JMHLW, Japanese Ministry of Health, Labor, and Welfare; patients with DIC scores of ≥7 points, 5–6 points, and ≤4 points were diagnosed with JMHLW-DIC, JMHLW-Pre-DIC, and JMHLW-Non-DIC; ISTH, International Society of Thrombosis Hemostasis; JAAM, Japanese Association for Acute Medicine; mJAAM, modified JAAM; DIC (≥3), more than 3 points of mJAAM DIC score; DIC (≥4), more than 4 points of mJAAM DIC score; *** *p* < 0.001; ** *p* < 0.01; NS, not significant.

The DIC index was significantly higher in patients with JMHLW-DIC (6.10; 2.73–13.1) than in those with JMHLW-Pre-DIC (1.44; 0.74–2.10) and higher in those with JMHLW-Pre-DIC than in JMHLW-Non-DIC (0.09; 0.03–0.27). The DIC index was also significantly higher in those with ISTH-DIC (6.49; 3.30–14.78) than in those with ISTH-Non-DIC (0.11; 0.04–0.37), and higher in those with mJAAM-DIC (≥4) (3.48; 1.97–7.33) and mJAAM-DIC (≥3) (1.91; 0.93–4.09) than in those with mJAAM-Non-DIC (0.09; 0.03–0.24) (Figure 2). Although there was no significant difference in the DIC index between those with JMHLW-DIC and those with ISTH-DIC, the DIC index was significantly higher in those with JMHLW-DIC and ISTH-DIC than in those with mJAAM DIC (≥4) or mJAAM DIC (≥3).

ROC analyses showed that the diagnostic agreement was the highest for the JMHLW score among the three diagnostic criteria; after this, in order, mJAAM DIC (≥4), ISTH-DIC, JMHLW-Pre-DIC, and mJAAM DIC (≥3) showed the highest diagnostic agreement (Table 6).

Regarding the relationship between DIC and outcome, the PT-INR, D-dimer level, DIC index, JMHLW-DIC score, ISTH-DIC, and mJAAM-DIC scores were significantly higher in non-survivors than in survivors, and their platelet counts were significantly lower (Table 7). ROC analyses showed that the PT-INR, D-dimer level, platelet count, DIC index, JMHLW-DIC, ISTH-DIC, and mJAAM-DIC scores were related to the outcomes. The cutoff values of the PT-INR, platelet count, and D-dimer for diagnosing DIC were associated with different outcomes. The cutoff values of the DIC index, JMHLW-DIC, ISTH-DIC, and mJAAM scores were lower for outcome than for diagnosing using JMHLW-DIC. AUC was 0.712–0.794 for PT-INR, platelet count, and D-dimer, and 0.815–0.869 for the DIC index and three diagnostic criteria (Table 8).

## 4. Discussion

The utility of many laboratory parameters, such as the PT level [18,19], platelet count [18,19], D-dimer level [19], fibrinogen level [18,19], FDP level [18,20], antithrombin level [21,34], tissue factor (TF) level [35,36], and sCLEC-2 level [29,37] has been proposed for the diagnosis of DIC. Among these items, the PT and D-dimer levels and platelet counts are common and routine laboratory tests, with most physicians using PT and D-dimer levels and platelet counts worldwide. PT is useful for monitoring warfarin [38], evaluating the function of the liver [39], and evaluating DIC [40,41]. Thrombocytopenia is caused by a low production of platelets, such as that observed in aplastic anemia [42,43], the consumption of platelets due to disseminated micro-thrombosis such as DIC [44,45], or thrombotic microangiopathy [46,47]. The D-dimer level is useful for diagnosing venous thromboembolism [48,49] and DIC [50,51]. In addition, the PT-INR, platelet count, and FDP have been correlated with the JMHLW DIC score, with moderate agreement with JMHLW DIC based on ROC analyses. Therefore, our finding that the DIC index (formula “PT-INR × D-dimer/platelet count”) was correlated with the JMHLW DIC score and strongly agreed with the JMHLW DIC (AUC = 0.995) was expected. PT-INR refers to the consumption clotting factor, platelet refers to the activation or consumption of platelets, and D-dimer refers to the formation and consumption of fibrins. Therefore, DIC, which refers to disseminated microthrombosis and coagulopathy, is further reflected in combination with PT-INR, platelet count, and D-dimer. Although our findings suggested that the D-dimer/platelet count ratio was sufficiently useful for diagnosing DIC. PT-INR could also be used for the diagnosis of pre-DIC. However, the pre-DIC state has not been well defined.

The AUC for the diagnosis of JMHLW DIC was 0.993 in the super formula using sCLEC-2 (sCLEC-2 × D-dimer/platelet count) [29], suggesting that the DIC index has a similar diagnostic capacity to the super formula using sCLEC-2 [29]. Although the super formula using sCLEC-2 is more useful for the diagnosis of DIC and pre-DIC, sCLEC-2 cannot be measured in general hospitals, suggesting that the DIC index may be more useful than a super formula using sCLEC-2. It has been reported [52,53,54] that the D-dimer/platelet ratio, which helps to discriminate preeclampsia from normal pregnancy and gestational hypertension [52], correlates with unfavorable outcomes in hepatitis B virus-related decompensated cirrhosis [53] and is predictive of the severity, ICU admission, and mortality of COVID-19 patients [54]. However, there are few reports on the utility of the PT/platelet ratio, PT-INR × D-dimer level, or PT-INR × D-dimer level/platelet count for the diagnosis of DIC, except for a report on a super formula using sCLEC-2 [29]. The AUC of the ROC analyses of the DIC index for DIC diagnosed using the three diagnostic criteria was highest for JMHLW-DIC, followed in order by m JAAM-DIC (≥4), ISTH-DIC, JMHLW-Pre-DIC, and m JAAM-DIC (≥3); this suggests that the DIC index may be useful for the diagnosis of advanced DIC.

The PT-INR, D-dimer level, DIC index, JAHLW DIC score, and modified JAAM DIC score were significantly higher in non-survivors than in survivors, and their platelet count was significantly lower; this suggests that these biomarkers, the DIC index, and the DIC score were related to the outcome. ROC analyses showed that the AUC for outcome was not significantly high for the PT-INR, D-dimer level, and platelet count but was highest for the DIC diagnostic score and DIC index, suggesting that the prediction of outcomes is better when multiple biomarkers (DIC index) and DIC scoring systems are used. The cutoff value for outcomes was markedly lower for the DIC index and three DIC scores when compared for the diagnosis of DIC, indicating that the early treatment of DIC before its diagnosis using previously established diagnostic criteria may improve the outcome of DIC. The diagnosis of pre-DIC has been previously proposed [55,56,57], suggesting that the prophylaxis may be important for improving the outcome of DIC. Therefore, application of the SIC diagnostic criteria [24,58,59] could be employed in critical care.

In clinical practice, the modified JAMA (JAMA-2) without the SIRS score, which is the same as m JAAM-DIC (≥3), was proposed last year [60,61]. Although the diagnostic criteria for DIC may become simpler or easier, it requires scoring by physicians. Meanwhile, the DIC index can be automatically available in clinical records. In future clinical settings, due to warnings from the DIC index, clinicians will make a definitive diagnosis of DIC using the scoring system.

The limitations of this study include retrospective single-center design and the fact that SIRS data were missing for the JAAM criteria. In addition, this study included many non-DIC patients. Therefore, the biomarkers and diagnostic criteria for the DIC and DIC index may be overrated, requiring prospective validation in a multi-center cohort.

## 5. Conclusions

The DIC index was highly consistent with the three diagnostic criteria for DIC and its related outcomes. Although the DIC index and DIC score were related to the outcome, the cutoff value for predicting the outcome was markedly lower than that for diagnosing DIC. Therefore, a prospective validation in a multi-center cohort using DIC index is needed.

## Figures and Tables

**Table 1:** Diagnostic criteria for DIC for JMHLW, ISTH-DIC and mJAAM.

DIC	JMHLW-DIC	ISTH-DIC	mJAAM-DIC
PT-INR, PT (s)	≥1.25, 1 P ≥1.67, 2 P	≥3 s, 1 P ≥6 s, 2 P	≥1.2, 1 P
FDP (mg/L)	≥10, 1 P ≥20, 2 P ≥40, 3 P		≥10, 1 P ≥25, 3 P
D-dimer (mg/L)		≥5, 2 P ≥10, 3 P	
Fibrinogen (g/L)	≤1.5, 1 P ≤1.0, 2 P	≤1.0, 1 P	
Platelet count (×10^10^/μL)	≤12, 1 P ≤ 8, 2 P ≤ 5, 3 P	≤10. 1 P ≤5, 2 P	≤12, 1 P ≤8, 3 P
Bleeding symptom	Positive, 1 P		
Organ failure	Positive, 1 P		
DIC	≥7 P	≥5 P	≥3 or 4 P

DIC, disseminated intravascular coagulation; JMHLW, the Japanese Ministry of Health, Labor, and Welfare; ISTH, the International Society of Thrombosis Hemostasis, JAAM, the Japanese Association for Acute Medicine; mJAAM, modified JAAM; PT, prothrombin time; INR, international normalized ratio; FDP, fibrinogen and fibrin degradation products.

**Table 2:** Subjects with JMHLW-DIC, JMHLW-Pre-DIC, or JMHLW-Non-DIC.

JMHLW DIC Criteria	JMHLW-DIC	JMHLW- Pre-DIC	JMHLW-Non-DIC
Age (years)	74 (66–84)	77 (60–82)	71 (55–82)
Sex (F:M)	21:51	45:67	601:715
Infectious disease	30	47	212
Digestive system disease	1	2	85
Chest or abdominal aneurysm	3	8	75
Hematological malignancy	1	2	64
Trauma	6	7	54
Obstetrics disease	2	1	48
Respiratory disease	1	3	34
Arrhythmia	2	1	18
Thrombosis of peripheral artery	1	1	14
Heat illness	1	1	33
Cardiac pulmonary arrest	21	14	32
Solid cancer	3	4	29
Acute coronary syndrome	0	5	67
Heart failure	0	5	81
Convulsive disorder	0	1	25
Metabolic and endocrine diseases	0	1	19
Venous thromboembolism	0	2	41
Cerebral bleeding	0	5	53
Non-malignant hematological disease	0	0	38
Cerebral thrombosis	0	0	145
Indefinite compliant syndrome	0	0	88
Others	0	0	61
Total	72	112	1316

Age is shown as the median (25th–75th percentile). DIC, disseminated intravascular coagulation; JMHLW, Japanese Ministry of Health, Labor, and Welfare; the DIC score is based on the JMLHW diagnostic DIC score; patients with DIC scores of ≥7 points, 5–6 points, and ≤4 points were diagnosed with JMHLW-DIC, JMHLW-Pre-DIC, and JMHLW-Non-DIC, respectively.

**Table 3:** DIC index.

Parameter	Evaluation
PT-INR	Consumption of coagulation factors
1/Platelet count	Activation and consumption of platelet
D-dimer	Fibrin formation and degradation
PT-INRxD-dimer/Platelet count	Disseminated microvascular coagulation

DIC, disseminated intravascular coagulation; PT, prothrombin time; INR, international normalized ratio.

**Table 4:** Correlation of the JMHLW-DIC score with PT-INR, 1/Platelet count, D-dimer levels, and DIC index.

Parameter	r	Formular	*p*
PT-INR	0.449	Y = −0.226 + 1.814X	*p* < 0.001
1/Platelet	0.159	Y = 0.049 + 0.015X	*p* < 0.001
D-dimer	0.542	Y = −1.494 + 5.289X	*p* < 0.001
DIC index	0.192	Y = −7.084 + 6.021X,	*p* < 0.001

PT, prothrombin time; INR, international normalized ratio; DIC, disseminated intravascular coagulation; r, correlation coefficient; the DIC index is calculated using the following formula: DIC index = D-dimer × PT-INR/Platelet count.

**Table 5:** ROC analyses of the PT-INR, Platelet count, D-dimer, PT-INR/Platelet count, PT-INRxD- dimer, D-dimer/Platelet count, and DIC index.

JMHLW-DIC vs. JMHLW-Non-DIC				
Parameter	Cutoff	Sensitivity	AUC	Odds Ratio
PT-INR	1.21	82.2%	0.893	20.9
Platelet count	13.9	85.7%	0.910	36.7
D-dimer	10.1	92.5%	0.971	137
PT-INR/Platelet count	0.10	92.5%	0.965	134
PT-INRxD-dimer	13.3	93.3%	0.980	177
D-dimer/Platelet count	11.2	95.6%	0.990	1132
DIC index	1.216	97.2%	0.995	1138
JMHLW-DIC or -Pre-DIC vs. JMHLW-Non-DIC				
PT-INR	1.10	76.2%	0.828	9.88
Platelet count	16.3	75.8%	0.812	9.75
D-dimer	7.76	86.9%	0.930	44.3
PT-INR/Platelet count	0.07	80.2%	0.879	15.6
PT-INRxD-dimer	8.90	86.9%	0.938	43.7
D-dimer/Platelet count	0.49	89.1%	0.959	65.2
DIC index	0.59	90.2	0.964	87.1

DIC, disseminated intravascular coagulation; JMHLW, Japanese Ministry of Health, Labor, and Welfare; patients with DIC scores of ≥7 points, 5–6 points, and ≤4 points were diagnosed with JMHLW-DIC, JMHLW-Pre-DIC, and JMHLW-Non-DIC; ROC, receiver operating characteristic; AUC, area under the curve; PT, prothrombin time; INR, international normalized ratio.

**Table 6:** ROC analyses of the DIC index and the JMHLW, ISTH overt DIC, and modified JAAM score.

ROC Analyses	Cutoff	Sensitivity	AUC	Odds Ratio
JMHLW-DIC vs. JMHLW-non-DIC	1.216	97.2%	0.995	1138
JMHLW-DIC + Pre-DIC vs. JMHLW-non-DIC	0.590	90.2%	0.964	87.1
ISTH overt-DIC vs. non-ISTH-overt DIC	1.199	91.0%	0.979	228
mJAAM-DIC (score of ≥4) vs. non-mJAAM DIC	0.682	93.9%	0.984	240
mJAAM-DIC (score of ≥3) vs. non-mJAAM DIC	0.449	88.8%	0.954	54.0

DIC, disseminated intravascular coagulation; JMHLW, the Japanese Ministry of Health, Labor, and Welfare; ISTH, the International Society of Thrombosis Hemostasis; mJAAM, modified Japanese Association for Acute Medicine; non-mJAAM DIC, patients with mJAAM DIC score of ≤2; ROC, receiver operating characteristic; AUC, area under the curve.

**Table 7:** Outcome and parameters of the DIC index.

Outcome	Survivors	Non-Survivors	
PT-INR	1.02 (0.94–1.12)	1.28 (1.09–1.70)	*p* < 0.001
D-dimer (mg/L)	1.88 (0.71–6.81)	10.6 (3.5–29.0)	*p* < 0.001
Platelet count (1 × 10^10^)	20.0 (15.8–27.2)	14.4 (9.0–21.6)	*p* < 0.001
JMHLW-DIC score	1.0 (0.0–2.0)	5.0 (3.0–7.0)	*p* < 0.001
DIC index	0.002 (0.001–0.005)	0.976 (0.315–3.731)	*p* < 0.001
ISTH-DIC score	1.0 (0.0–2.0)	5.0 (3.5–7.0)	*p* < 0.001
mJAAM DIC score	1.0 (0.0–2.0)	5.0 (4.0–7.0)	*p* < 0.001

Data are shown as the median (25th–75th percentile). DIC, disseminated intravascular coagulation; PT, prothrombin time; INR, international normalized ratio; JMHLW, the Japanese Ministry of Health, Labor, and Welfare; ISTH, the International Society of Thrombosis Hemostasis; JAAM, the Japanese Association for Acute Medicine; mJAAM, modified JAAM; AUC, area under the curve.

**Table 8:** ROC analyses of parameters, DIC index, and three DIC scores for outcomes (non-survivors vs. survivors).

ROC Analyses	Cutoff	Sensitivity	AUC	Odds Ratio
PT-INR	1.10	72.9%	0.794	7.3
D-dimer	18.5	64.0%	0.712	3.3
Platelet count	5.0	69.7%	0.778	5.5
DIC index	0.33	74.2%	0.815	8.30
JMHLW-DIC score	2.0	77.3%	0.867	11.6
ISTH-DIC score	1.96	77.6%	0.869	13.5
mJAAM-DIC score	2.0	77.9%	0.868	12.3

Data are shown as the median (25th–75th percentile). DIC, disseminated intravascular coagulation; PT, prothrombin time; INR, international normalized ratio; JMHLW, the Japanese Ministry of Health, Labor, and Welfare; ISTH, the International Society of Thrombosis Hemostasis; JAAM, the Japanese Association for Acute Medicine; mJAAM, modified JAAM; AUC, area under the curve.

## Data Availability

The data presented in this study are available on request to the corresponding author. The data are not publicly available due to privacy restrictions.

## Abbreviations

The following abbreviations are used in this manuscript:

DIC	disseminated intravascular coagulation
SIC	sepsis-induced coagulopathy
TMA	thrombotic microangiopathy
SIRS	systemic inflammatory response syndrome
JMHLW	Japanese Ministry of Health, Labor, and Welfare
ISTH	International Society of Thrombosis Hemostasis
JAAM	Japanese Association for Acute Medicine
PT	prothrombin time
INR	international normalized ratio
FDP	fibrinogen and fibrin degradation products
sCLEC-2	soluble C-type lectin-like receptor 2
ROC	receiver operating characteristic
r	correlation coefficient
AUC	area under the curve
